# Validation of the capnodynamic method to calculate mixed venous oxygen saturation in postoperative cardiac patients

**DOI:** 10.1186/s40635-025-00741-z

**Published:** 2025-03-07

**Authors:** Mats Wallin, Magnus Hallback, Hareem Iftikhar, Elise Keleher, Anders Aneman

**Affiliations:** 1 Department of Physiology and Pharmacology, Karolinska Institute, Stockholm, Sweden; 2 Maquet Critical Care AB, Solna, Sweden; 3 Faculty of Medicine, University of New South Wales, Sydney, Australia; 4 Intensive Care Unit, Liverpool Hospital, South Western Sydney Local Health District, Sydney, Australia

**Keywords:** Capnometry, Pulmonary artery catheterisation, Mixed venous oxygen saturation, Cardiac output, Pulmonary blood flow

## Abstract

**Background:**

Cardiac output and mixed venous oxygen saturation are key variables in monitoring adequate oxygen delivery and have typically been measured using pulmonary artery catheterisation. The capnodynamic method measures effective pulmonary blood flow utilising carbon dioxide kinetics in ventilated patients. Combined with breath-by-breath measurements of carbon dioxide elimination, a non-invasive approximation of mixed venous oxygen saturation can be calculated.

**Methods:**

This study primarily investigated the agreement between mixed venous oxygen saturation calculated using the capnodynamic method and blood gas analysis of mixed venous blood sampled via a pulmonary artery catheter in 47 haemodynamically stable postoperative cardiac patients. Both measurements were synchronised and performed during alveolar recruitment by stepwise changes to the level of positive end-expiratory pressure. Simultaneously, we studied the agreement between effective pulmonary blood flow and thermodilution cardiac output. The Bland–Altman method for repeated measurements and calculation of percentage error were used to examine agreement. Measurements before and after alveolar recruitment were analysed by a paired t test. The study hypothesis for agreement was a limit of difference of ten percentage points between mixed venous oxygen saturation using the capnodynamic algorithm vs. catheter blood gas analysis.

**Results:**

Capnodynamic calculation of mixed venous saturation compared to blood gas analysis showed a bias of -0.02 [95% CI − 0.96–0.91] % and limits of agreement at 8.8 [95% CI 7.7–10] % and − 8.9 [95% CI -10–− 7.8] %. The percentage error was < 20%. The effective pulmonary blood flow compared to thermodilution showed a bias of − 0.41 [95% CI − 0.55–− 0.28] l.min^−1^ and limits of agreement at 0.56 [95% CI 0.41–0.75] l.min^−1^ and − 1.38 [95% CI − 1.57–-1.24] l.min^−1^. The percentage error was < 30%. Only effective pulmonary blood flow increased by 0.38 [95% CI 0.20–0.56] l.min^−1^ (p < 0.01) after alveolar recruitment.

**Conclusions:**

In this study, minimal bias and limits of agreement < 10% between mixed venous oxygen saturation calculated by the capnodynamic method and pulmonary arterial blood gas analysis confirmed the agreement hypothesis in stable postoperative patients. The effective pulmonary blood flow agreed with thermodilution cardiac output, while influenced by pulmonary shunt flow.

**Supplementary Information:**

The online version contains supplementary material available at 10.1186/s40635-025-00741-z.

## Introduction

Supporting adequate oxygen delivery is essential to the management of patients with cardiorespiratory compromise admitted to the intensive care unit. Cardiac output (CO) and mixed venous oxygen saturation (SvO_2_) are key physiological variables to monitor the systemic oxygen delivery and its sufficiency in relation to oxygen consumption. The clinical reference standards for measuring these variables remain pulmonary artery thermodilution (CO_TD_) and multi-wavelength oximetry blood gas analysis following insertion of a pulmonary artery catheter (PAC-SvO_2_). This invasive procedure has largely been replaced by other methods such as echocardiography and pulse contour analysis to assess CO, with SvO_2_ often ignored and replaced by markers of perfusion such as arterial lactate levels. A non-invasive alternative to pulmonary artery catheterisation that could provide similar information on systemic oxygenation with acceptable precision and accuracy would be of considerable clinical relevance and formed the impetus for this clinical validation study of the capnodynamic method.

The amount of exhaled carbon dioxide represents an integral of metabolism, ventilation and perfusion and is during steady state equal to carbon dioxide production, VCO_2_. By applying a controlled alternating breathing pattern in intubated, appropriately ventilator-synchronised patients, the capnodynamic method, based on a modification of the differential Fick’s principle [1, 2], enables continuous, non-invasive estimation of CO as represented by the effective pulmonary blood flow (EPBF). The EPBF amounts to the blood flow participating in gas exchange [3, 4] and is thus of particular interest since it is the non-shunted blood flow through the lungs that carries oxygenated blood to the systemic circulation. The primary measurement by the capnodynamic method, VCO_2_, may be extrapolated to VO_2_ if a fixed respiratory quotient is assumed. Previous experimental data support this inference of VO_2_ and the calculation of SvO_2_ using the capnodynamic method (CAPNO-SvO_2_) [5, 6]. While the correlations between CO_TD_ and EPBF and PAC-SvO_2_ and CAPNO-SvO_2_, respectively, have been reported in experimental settings [5–7], this report presents the first clinical study evaluating the performance of CAPNO-SvO_2_ in patients treated in ICU against PAC-SvO_2_ obtained from pulmonary artery blood samples. For the primary aim, we hypothesised that CAPNO-SvO_2_ and PAC-SvO_2_ would demonstrate a clinically relevant agreement in adult postoperative cardiac patients undergoing a lung recruitment manoeuvre. Based on previous experimental results [5, 6], an acceptable limit of difference between the methods was set at ten percentage points. We also investigated the agreement between EBPF and CO_TD_ as the secondary aim, but no numerical criterion was set for the agreement between these variables given their inherent physiological differences.

## Methods

This investigator initiated study was approved by the South Western Sydney Local Health District Human Research Ethics Committee (2020/ETH00778, 15th of April 2020) with waiver of written consent. Verbal information was provided to the person responsible of the patient. The general capnodynamic study protocol was published on Open Science Framework (https://osf.io/93r7n/?view_only=d4a8e76bc28b4ae6a1d642d4a756659b) on the 7th April 2020. The study is reported using the STARD 2015 guidelines [8, 9] (see Additional file 1, Table S1). The patient characteristics and clinical procedures in this study have previously been described in detail in a report of capnodynamic variables in relation to alveolar recruitment and oxygen delivery [10]. Patients were eligible for inclusion if haemodynamically stable and the treating clinical team agreed to PEEP titration for potential alveolar recruitment, a pulmonary arterial catheter had been inserted intraoperatively and mechanical ventilation with full patient-ventilator synchrony was expected to remain for at least another two hours. Unstable patients were excluded from the study as suggested by any postoperative bleeding with imminent return to operating theatres considered, ongoing or imminent need for mechanical circulatory support, or haemodynamic instability (> 50% increase in vasopressor administration after fluid loading in the first hour or noradrenaline > 0.3 µg kg^−1^ min^−1^). Patients were sedated (propofol, fentanyl) with lung ventilation as per institutional protocol using a volume-controlled mode (Draeger V500, Draeger, Lubeck, Germany), a positive end-expiratory pressure (PEEP) of 5 cm H_2_O, a respiratory rate (RR) of 12 min^−1^ with an inspiratory-to-expiratory ratio of 1:2, tidal volume (TV) 6–8 ml kg^−1^ ideal body weight, F_i_O_2_ initially set at 0.5 and then titrated to a S_p_O_2_ > 95%. Alveolar recruitment was performed at the discretion of the treating team by a stepped incremental PEEP manoeuvre.

### Capnodynamic method

Before study procedures the endotracheal tube was briefly clamped and the Draeger ventilator switched to a modified research Servo-I ventilator (Maquet Critical Care, Solna, Sweden) with the F_i_O_2_, TV, PEEP and ventilation mode unchanged. The research Servo-I ventilator used a customised breathing pattern with short expiratory holds to three out of nine consecutive breaths to produce recurring changes in the alveolar partial pressure of CO_2_ of at least 0.4 kPa. The RR was adjusted to maintain the same minute ventilation as before changing to the research ventilator. The capnodynamic method is a further development of the differential Fick method for measurement of CO. It continuously, breath by breath, measures non-shunted, i.e. effective pulmonary blood flow (EPBF) and its algorithm has been described in detail elsewhere [10–12]. For each breath in the sequence of nine breaths in the period ventilation pattern, a dynamic model equation for alveolar CO2 concentration is formulated that contains known measured quantities and three unknown parameters (EPBF, lung volume, venous CO2 partial pressure). The unknown parameters are determined so that the equations are satisfied in a least-square-error sense.

The primary measurement from the capnodynamic method, an accurate breath-by-breath determination of VCO_2_, is also one of the main variables of the respiratory exchange ratio during controlled mechanical ventilation. The ratio between carbon dioxide elimination (VCO_2_) and oxygen uptake (VO_2_), is equal to the cellular respiratory quotient (RQ) in resting equilibrium, which forms the basis for indirect calorimetry. A predetermined RQ of 0.85 based on reports for postoperative ICU patients [13, 14] was used in this study to calculate VO_2_. The following equations describe the derivation of CAPNO-SvO_2_. All gas volumes related to exchange rate (VCO_2_ and VO_2_) and blood gas contents are referred to the gas reference standard temperature and pressure (STP, 0 °C and 101.3 kPa).

1 $${\text{VO}}_{2}=\frac{{\text{VCO}}_{2}}{\text{RQ}}$$

where VO_2_ is oxygen consumption (ml_STP_.min^−1^), VCO_2_ is CO_2_ production rate (ml_STP_.min^−1^), and RQ is the respiratory quotient (set to 0.85 in this study). The capnodynamic algorithm (Matlab®, Mathworks, Natick, MA, USA), running on a dedicated laptop connected to the ventilator, was used to determine the VCO_2_ from each breath’s volumetric capnogram created in real time by synchronizing the data from a mainstream capnometer (Capnostat 3®, Philips Respironics, Philadelphia, PA, USA) with the data from the ordinary flow transducer in the Servo-I ventilator. A 20-min moving mean value of the breath-by-breath determination of VCO_2_ was entered in Eq. 1 to reduce temporary fluctuations in CO_2_ elimination and to provide stable VCO_2_ measurements reflecting the underlying metabolism.

2 $$\text{EPBF}=\frac{{\text{VO}}_{2}}{{\text{CcO}}_{2}-{\text{CvO}}_{2}}$$

This is the Fick’s equation for oxygen transfer in the lung where EPBF is the effective pulmonary blood flow (l.min^−1^) participating in gas exchange, VO_2_ is oxygen consumption (ml_STP_.min^−1^), CcO_2_ is pulmonary end capillary oxygen content (ml_STP_.l^−1^), and CvO_2_ is pulmonary mixed venous oxygen content (ml_STP_.l^−1^).

Equation 2 can be rearranged for CvO_2_:

$${\text{CvO}}_{2}={\text{CcO}}_{2}-\frac{{\text{VO}}_{2}}{\text{EPBF}}$$

The contents of O_2_ in capillary (CcO_2_) and mixed venous blood (CvO_2_) (ml_STP_.l_blood_^−1^) are given by:

$${\text{CcO}}_{2} = {\upalpha } \cdot {\text{PcO}}_{2} + {\text{C}}_{{\text{H}}} \cdot {\text{Hb}} \cdot {\text{ScO}}_{2}$$

$${\text{CvO}}_{2} = {\upalpha } \cdot {\text{PvO}}_{2} + {\text{C}}_{{\text{H}}} \cdot {\text{Hb}} \cdot {\text{SvO}}_{2}$$

where PcO2 is pulmonary end capillary partial pressure of O2 (kPa), PvO2 is mixed venous partial pressure of O2 (kPa), ScO2 is pulmonary end capillary oxygen saturation (fraction), and SvO2 is mixed venous oxygen saturation (fraction). The solubility constant for O2 in blood plasma α is 0.224 (mlSTP l−1 kPa), the maximum oxygen carrying capacity of haemoglobin (Hüfner constant CH) is 1.35 (mlSTP.g−1) and Hb represents the haemoglobin content in blood (g.l−1). The oxygen partial pressure of end-capillary blood was assumed to be in equilibrium with the alveolar gas, with the partial pressure of oxygen calculated from the ventilator FiO2 and the alveolar gas equation using the assumed value of RQ instead of the respiratory exchange ratio.

$${\text{PAO}}_{{2}} \, = \,{\text{F}}_{{\text{i}}} {\text{O}}_{{2}} \left( {{\text{P}}_{{{\text{atm}}}} - {\text{P}}_{{{\text{H2O}}}} } \right) - {\text{PACO}}_{{2}} ({1} - {\text{F}}_{{\text{i}}} {\text{O}}_{{2}} \left( {{1}{-}{\text{RQ}}} \right))/{\text{RQ}}$$

The CAPNO-SvO_2_ was derived from CvO_2_ in Eq. 2 by employing the oxygen content equations:

3 $${\text{CAPNO - SvO}}_{2} = {\text{ScO}}_{2} - \frac{{{\text{VCO}}_{2} }}{{{\text{RQ}} \cdot {\text{C}}_{{\text{H}}} \cdot {\text{Hb}} \cdot {\text{EPBF}}}} + \frac{{\upalpha }}{{{\text{C}}_{{\text{H}}} \cdot {\text{Hb}}}}\left( {{\text{PcO}}_{2} - {\text{PvO}}_{2} } \right)$$

where $\langle {\text{VCO}}_{2}\rangle$ denotes the 20-min moving average of the measured CO_2_ elimination. Based on a sigmoid relation between oxygen saturation and partial pressure, the solution is obtained from an iterative mathematical procedure as previously described in detail in experimental reports, thus creating breath-by-breath estimations of SvO_2_ [5].

### Study protocol

After 20 min of baseline ventilation and before the recruitment manoeuvre (PRE_RM_), a mixed venous blood sample was obtained from the distal port of the PAC after discarding the waste volume. Each sample was stored at room temperature and analysed as quickly as possible at every PEEP level by CO-oximetry (GEM Premier 5000, Artarmon, New South Wales, Australia) representing the clinical standard reference PAC-SvO_2_. Immediately after obtaining the PAC-SvO_2_ blood sample, pulmonary arterial thermodilution injections using 10 mL of room temperature saline were performed with the average of triplicate measurements within ± 10% representing the clinical standard reference CO_TD_ (Philips IntelliVue MX850, Philips, North Ryde, New South Wales, Australia). The PEEP was then increased by 5 cmH_2_O for 15 min followed by another increase of 5 cmH_2_O for 15 min with a complete set of measurements performed at the end of each PEEP level. The PEEP was then returned to the baseline level and a final set of measurements were performed 15 min after the recruitment manoeuvre (POST_RM_). Thus, four measurements of CAPNO-SvO_2_/PAC-SvO_2_ and EPBF/CO_TD_ were made for each patient. The patient was reconnected to the standard Draeger ventilator at the completion of study procedures.

### Data handling and statistical analyses

De-identified clinical and capnodynamic data were uploaded on a secure cloud server for off-line analyses with synchronisation to the continuous capnodynamic data output, using Microsoft Excel, version 16.46 for Mac, 2021, for data management. First, at the end of each PEEP-level, sequences of 27 breaths (3 cycles of 9) from periods of adequate patient-ventilator synchrony were selected from the capnodynamic data file and averaged. The investigator performing the off-line analyses of capnodynamic data was blinded to the clinical reference standard results, PAC-SvO_2_ and CO_TD_. Then, the timepoints of these capnodynamic measurements were compared with the timepoints for the CO_TD_ and PAC-SvO_2_ measurements. Only measurements pairs collected within 5 min of each other were accepted. No imputations were made for single missing data and measurements outside the time window were discarded from further analyses.

The details of statistical considerations are provided in the Additional file 1, Figures S1-S8. The Bland–Altman method for repeated measurements including a function to allow for the values to vary within subjects [15, 16] was used to examine the agreement of absolute values between paired recordings of CAPNO-SvO_2_ and PAC-SvO_2_ (%, percentage points) as well as EPBF and CO_TD_ (l.min^−1^). The normality distribution, homogeneity of variance and proportional bias were assessed prior, with adjustments made to the Bland–Altman analyses as appropriate. Bias, representing the mean difference between the investigated methods and the reference method, together with limits of agreement and their 95% CI are reported. Correlations are reported by Pearson correlation coefficient (r) including the 95% CI.

The mean percentage error was calculated as 1.96 × SD of the differences between the tested method and the reference method, divided by the mean of the reference method [17]. The effect of the recruitment manoeuvre was evaluated by a paired t-test of PRE_RM_ and POST_RM_ and reported as mean difference including the 95% CI. Inter-individual changes during the alveolar recruitment manoeuvre were analysed by repeated measurements ANOVA and intraindividual changes reported by variance and coefficient of variation.

A power calculation for the Bland–Altman analysis aimed to detect at a 95% confidence level that 80% of observations would lie within the hypothesized difference of ten percentage points with a power > 0.9 estimated that 191 data pairs were needed (see Additional file 1, Figure S1).

Analyses were performed in RStudio (version 2023.06.0 + 421), with statistical significance set at a two-sided *p*-value of < 0.05.

## Results

**Fig. 1:**
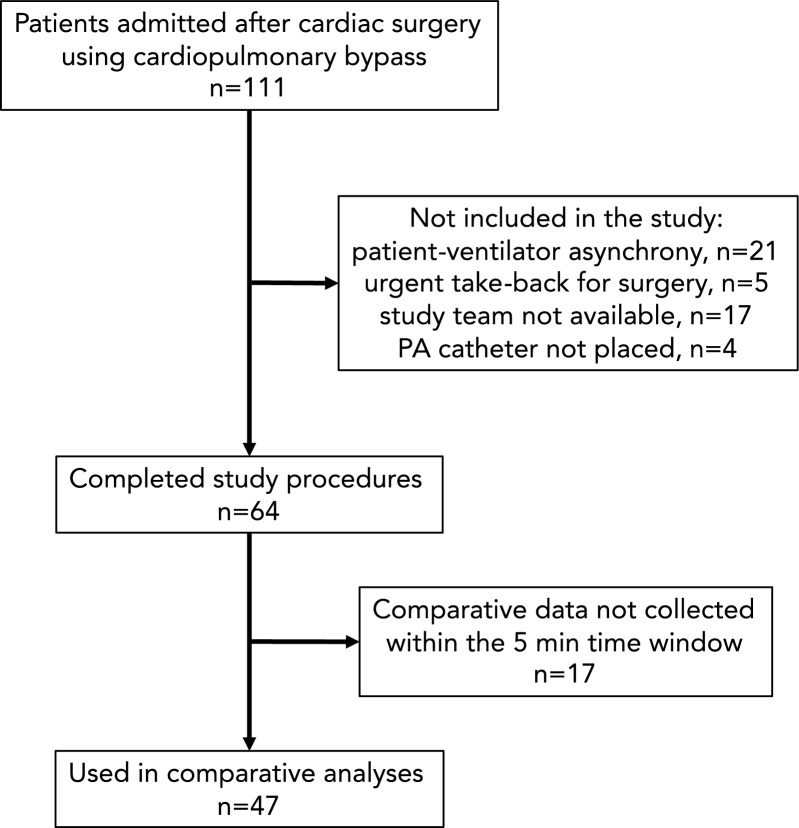
Recruitment flow diagram showing the inclusion (downward arrow) and exclusion (rightward arrow) of patients in this prospective, observational cohort study of post-cardiac surgical patients

The patient flow in the study is shown in Fig. 1. Forty-seven patients met all study eligibility criteria and a total of 185 data pairs were included in the statistical analyses (three missing pairs for POST_RM_). No data were discarded because of poor quality**.** Patients were 65 ± 11 years, predominantly male (68%) with coronary artery bypass grafting the most common procedure (64%) and had a SOFA score of 10 [9–12] on the day of admission. Full patient demographics are reported in Table 1.

**Table 1:** Patient characteristics (n = 47)

Age; years	65 ± 11
Weight; kg	83 ± 22
Height; cm	164 ± 17
Male sex; %	32 (68)
Surgical Procedure; %	
CABG Valve CABG + valve Thoracic aorta	30 (64) 11 (23) 5 (11) 2 (4)
By-pass time; min	108 ± 54
Cross clamp time; min	84 ± 42
APACHE III	50 (40–57)
SOFA	10 (9–12)
EuroScore II; %	1.9 (0.8–2.5)
ICU LOS; days	3.8 (2.0–4.4)
Hospital LOS; days	12 (7.61–20)

Values are mean ± standard deviation, number (proportion, %) or median (interquartile range)

*CABG* coronary artery bypass grafting, *APACHE* acute physiology and chronic health evaluation, *SOFA* sequential organ failure assessment, *LOS* length of stay

**Fig. 2:**
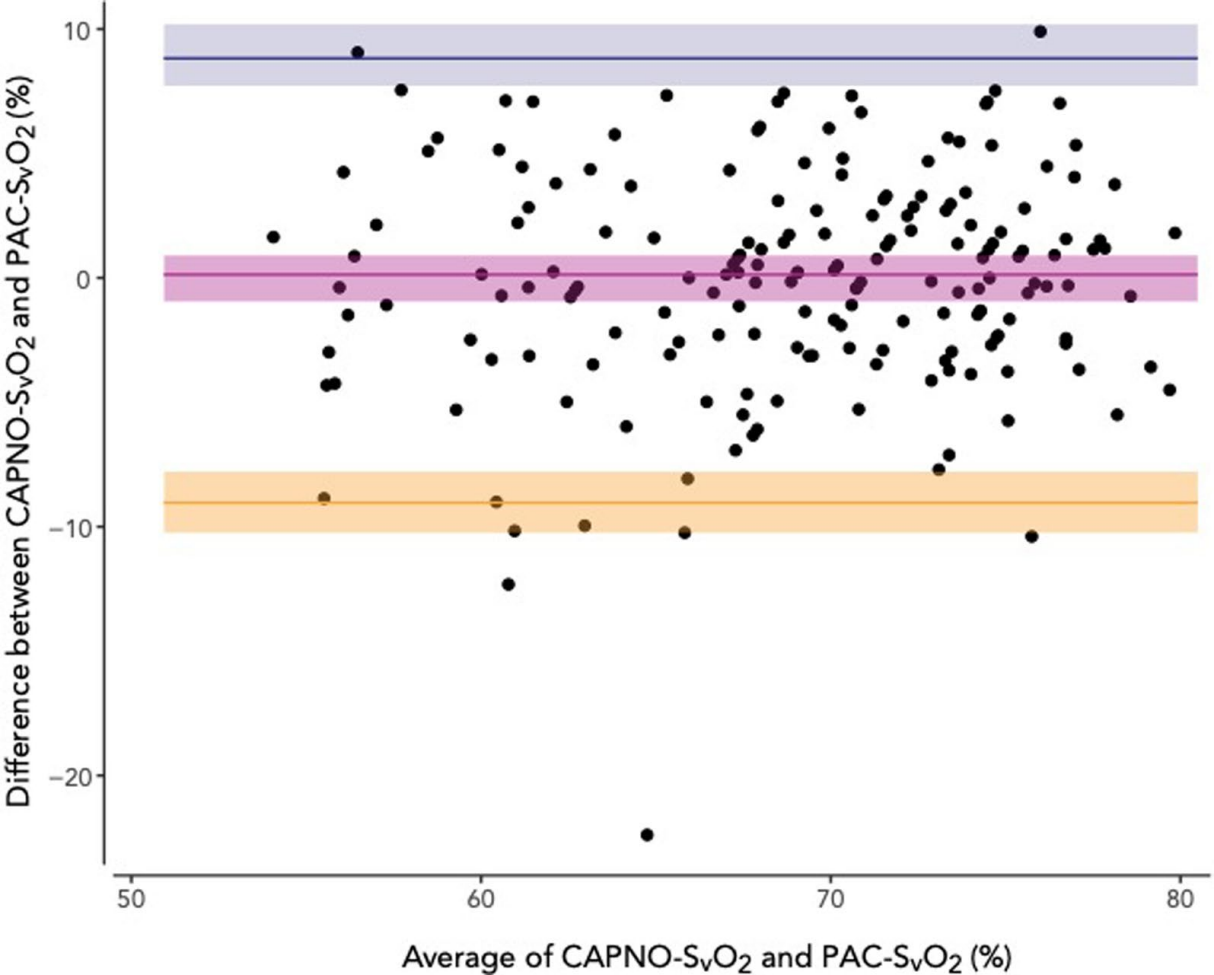
Repeated measurements Bland–Altman plot of capnodynamic calculation of mixed venous oxygen saturation (CAPNO-SvO_2_) vs. CO-oximetry of blood sample obtained via a pulmonary artery catheter (PAC-SvO_2_). The purple band indicates the bias and 95% CI, the blue band indicates the upper limit of agreement with 95% CI and the yellow band indicates the lower limit of agreement with 95% CI

**Fig. 3:**
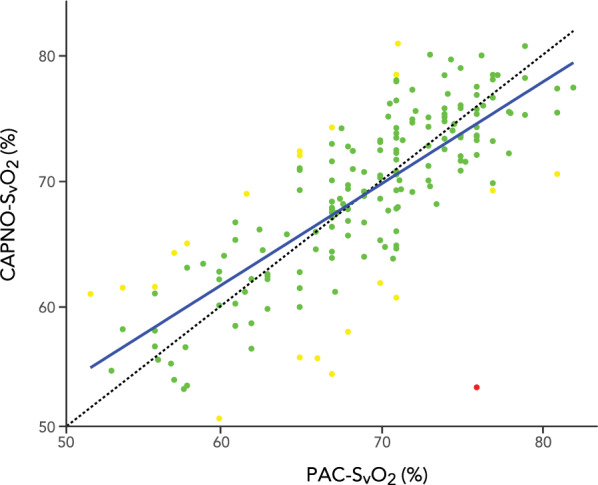
Correlation between capnodynamic calculation of mixed venous oxygen saturation (CAPNO-SvO_2_) vs. CO-oximetry of blood sample obtained via a pulmonary artery catheter (PAC-SvO_2_). The dotted line indicates the line of identity. Green dots represent data pairs with < 10% relative difference, yellow dots represent data pairs with ≥ 10% but < 20% relative difference, and the red dot represents one data pair with ≥ 20% relative difference between results

One data pair was identified in the CAPNO-SvO_2_ vs. PAC-SvO_2_ comparison as an outlier (23 percentage points difference with the PAC-SvO_2_ deviating, see Additional File 1, Figure S4) with no reason for the deviation evident from clinical and study documentation. These data were retained in the study analyses with normality rejected (p = 0.003, see Additional File 1, Figure S5) and a non-parametric repeated measures Bland–Altman analysis was performed. The bias between CAPNO-SvO_2_ and PAC-SvO_2_ was − 0.02 [95% CI − 0.96–0.91] % with the upper limit of agreement at 8.8 [95% CI 7.7–10] % and the lower limit of agreement at −8.9 [95% CI − 10–− 7.8] % (Fig. 2). The correlation coefficient between CAPNO-SvO_2_ and PAC-SvO_2_ was r = 0.77 (95% CI 0.70–0.82, p < 0.0001) (Fig. 3). Twenty data pairs (11% of all data pairs) demonstrated a relative difference for CAPNO-SvO_2_ ≥ 10% of PAC-SvO_2_ and one data pair (0.5% of all data pairs) demonstrated a relative difference ≥ 20% (Fig. 3). The descriptive statistics including bias and percentage errors for CAPNO-SvO_2_ and PAC-SvO_2_ throughout the recruitment manoeuvre are reported in Table 2. The percentage error was consistently < 20%. The variance and coefficients of variation were similar between CAPNO-SvO_2_ and PAC-SvO_2_, as well as between EPBF and COTD (see, file 1 Table S2). A sensitivity analysis excluding the outlier with a parametric Bland–Altman analysis demonstrated slightly narrower limits of agreement (see Additional file 1, Figure S2).

**Table 2:** Descriptive statistics, bias and percentage error comparing the capnodynamic mixed venous oxygen saturation (CAPNO-SvO_2_) and effective pulmonary blood flow (EPBF) versus blood gas analysis (PAC-SvO_2_) and thermodilution cardiac output (CO_TD_) using the pulmonary artery catheter (PAC)

	CAPNO-SvO_2_ (mean ± SD, %)	PAC-SvO_2_ (mean ± SD, %)	Bias (%)	PE	EPBF (mean ± SD, l.min^−1^)	CO_TD_ (mean ± SD, l.min^−1^)	Bias (l.min^−1^)	PE (%)
PRE_RM_	68.8 ± 7.1	69.4 ± 5.6	− 0.61	16%	3.90 ± 0.91	4.46 ± 0.93	− 0.55	20
PRE_RM_ + 5 cm H_2_O	68.7 ± 6.4	69.2 ± 6.8	− 0.47	10%	3.90 ± 0.80	4.32 ± 0.90	− 0.41	26
PRE_RM_ + 10 cm H_2_O	68.9 ± 6.7	67.3 ± 6.4	1.6	12%	3.95 ± 0.85	4.27 ± 0.92	− 0.31	25
POST_RM_ = PRE_RM_	69.6 ± 7.1	70.4 ± 6.5	− 0.77	11%	4.26 ± 1.0	4.65 ± 1.2	− 0.38	23

*PRE*_*RM*_ PEEP level prior to alveolar recruitment manoeuvre, *POST*_*RM*_ PEEP level after alveolar recruitment manoeuvre, *SD* standard deviation, *PE* percentage error

**Fig. 4:**
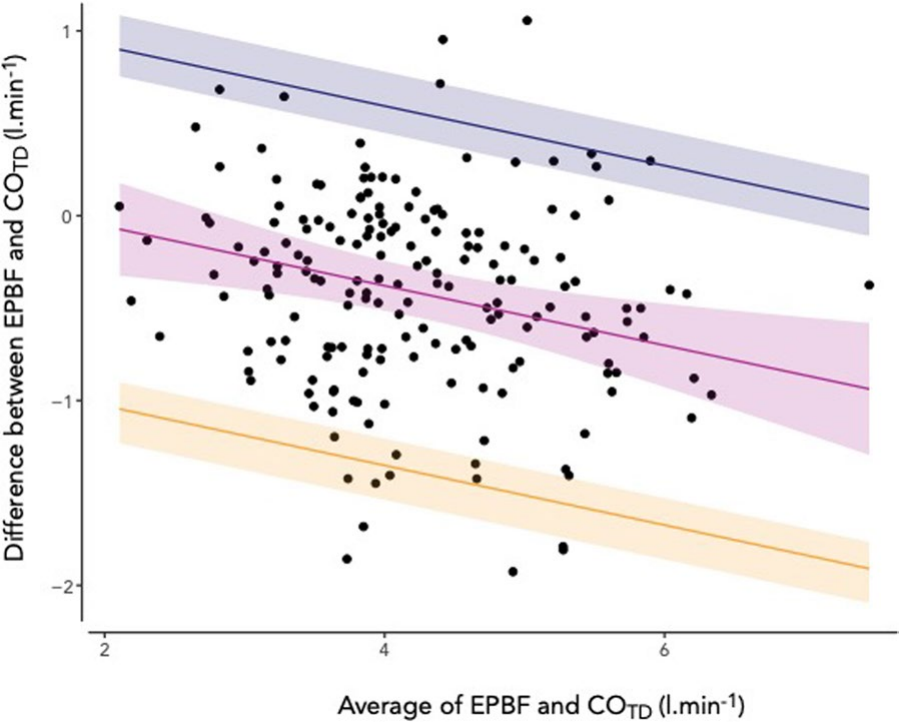
Repeated measurements Bland–Altman plot of capnodynamic measurement of effective pulmonary blood flow (EPBF) vs. thermodilution cardiac output using a pulmonary artery catheter (CO_TD_). The purple band indicates the bias and 95% CI, the blue band indicates the upper limit of agreement with 95% CI and the yellow band indicates the lower limit of agreement with 95% CI. The negative slopes indicate proportional bias reflecting the exclusion of pulmonary shunt flow in the EPBF measurement

**Fig. 5:**
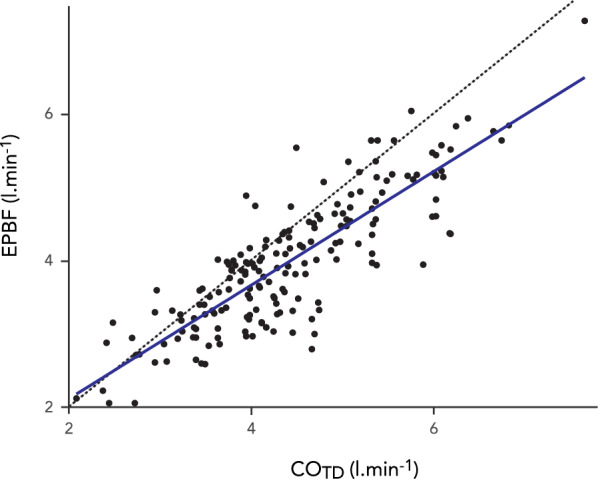
Correlation between capnodynamic measurement of effective pulmonary blood flow (EPBF) vs. thermodilution cardiac output using a pulmonary artery catheter (CO_TD_). The dotted line indicates the line of identity. No predefined relative difference criteria were used (cf. Figure 3) for these variables given their inherent physiological differences (cf. Figure 4)

The differences between CO_TD_ and EPBF demonstrated a proportional bias (p = 0.002) and the Bland–Altman method adjusted accordingly (see Additional file 1, Figure S8). The bias in the Bland–Altman analysis was −0.41 [95% CI − 0.55–− 0.28] l.min^−1^ with the upper limit of agreement at 0.56 [95% CI 0.41–0.75] l.min^−1^ and the lower limit of agreement at − 1.38 [95% CI − 1.57–− 1.24] l.min^−1^ (Fig. 4). The correlation coefficient was *r* = 0.85 (95% CI 0.80–0.88, p < 0.0001) (Fig. 5). The descriptive statistics including bias and percentage errors for EPBF and COTD throughout the recruitment manoeuvre are reported in Table 2. The percentage error between EPBF and CO_TD_ was consistently < 30% during the recruitment manoeuvre.

The EPBF (mean change 0.38 [95% CI 0.20–0.56] l.min^−1^, *p* < 0.01, ANOVA *p* = 0.01) increased after the recruitment manoeuvre, while no significant changes were observed in CAPNO-SvO_2_ (mean change 1.2 [95% CI − 0.13–2.2] %, *p* = 0.08, ANOVA *p* = 0.56), PAC-SvO_2_ (mean change 1.3 [95% CI − 0.32–2.9] %, *p* = 0.11, ANOVA *p* = 0.29) or CO_TD_ (mean change 0.21 [95% CI −0.03–0.44] l.min^−1^, *p* = 0.09, ANOVA p = 0.46) (see Table 2 and Additional file 1, Figure S9 for details).

## Discussion

This clinical validation study demonstrated, for its primary aim, an agreement between CAPNO-SvO_2_ and a contemporary blood sample from the pulmonary artery (PAC-SvO_2_) analysed with a CO-oximeter in haemodynamically stable postoperative cardiac patients. The bias was close to zero and the limits of agreement were < 10%, thus falling within the hypothesised clinically acceptable range. The secondary aim of comparing EPBF with thermodilution cardiac output showed a proportional bias of − 0.4 l.min^−1^ and wider limits of agreement, reflecting the importance of pulmonary shunt flow.

### Mixed venous oxygen saturation by the capnodynamic method.

The mixed venous oxygen saturation provides important information about oxygen delivery relative to oxygen needs and aids clinical decisions for critically ill patients. In a large observational cohort of postoperative cardiac patients monitored with a pulmonary artery catheter, low (< 60%) SvO_2_ on admission to ICU or persisting 4 h postoperatively was associated with increased 30-day and 1-year mortality [18] as well as early postoperative multiorgan dysfunction [19].

This study used Bland–Altman methodology to investigate the agreement between the noninvasive CAPNO-SvO_2_ and the invasively obtained PAC-SvO_2_, adhering to detailed reporting recommendations [20]. The bias for CAPNO-SvO_2_ compared to PAC-SvO_2_ was minimal and similar to previous animal studies [5, 6], supporting its accuracy. The limits of agreements were also comparable and within the predefined < 10% limits, indicating that the precision for CAPNO-SvO_2_ is consistent across both animal and clinical studies. The percentage error was 10–16% throughout the recruitment manoeuvre, demonstrating high precision of the agreement regardless of the level of PEEP. These results suggest that the non-invasive and nearly continuous CAPNO-SvO_2_ is comparable to the invasive and intermittent PAC-SvO_2_ based on the hypothesised agreement limits and the minimal bias.

The PAC-SvO_2_ used as a reference in this study may have an inherent measurement error related to obtaining and analysing blood samples under real-life clinical conditions. Insufficient sample mixing, air contamination in the syringe and an insufficient sluice volume withdrawn from the pulmonary artery catheter before the sample was taken may have introduced errors in the reference test [21]. Although blood samples were analysed as quickly as practically possible, typically within 5 min, they were not placed in ice or on a rotary shaker which is recommended for optimal results [21]. These limitations to PAC-SvO_2_ as the clinical reference are important to consider as the observed limits of agreement depend on the inherent precision of both methods.Repeated measurements during haemodynamically stable baseline conditions in previous experimental studies [5, 6] demonstrated inherent precisions of 5% points for CAPNO-SvO_2_ and 4% points for PAC-SvO_2_. As a result, a true SvO_2_ value of 69% can by chance be presented as 64% by one method and as 73% by the other. The difference of 9% points generated by this random variation informed the limits of agreement criterion of 10% points that formed part of the hypothesis for this agreement study. A reference method with smaller inherent measurement error might have narrowed the limits of agreement for CAPNO-SvO_2_.

The calculation of CAPNO-SvO_2_ requires, in addition to EPBF, an estimate of oxygen consumption (VO_2_), which was obtained from the measured VCO_2_ and a set, defined value for the RQ. Measuring VO_2_ in respiratory gases is challenging and has limited the use of indirect calorimetry in the perioperative clinical setting [13, 22]. A more practical way to determine RQ in the ICU is to calculate it from the nutritional energy distribution based on the proportion of fat, protein and carbohydrate administered [14]. Previous studies of fasting subjects [23] and postoperative patients in the ICU [13, 14] have shown that RQ is typically about 0.85, whether measured by indirect calorimetry or calculated from nutrient content [14]. An incorrectly set RQ would affect the bias compared to PAC-SvO_2_. Using a higher RQ of 0.9 (instead of 0.85) in this study would have increased the bias between CAPNO-SvO_2_ and PAC-SvO_2_ by 2% points. The negligible bias and acceptable limits of agreement demonstrated in this study with PAC-SvO_2_ corroborate, however, that the chosen RQ of 0.85 was appropriate for the study cohort of haemodynamically stable postoperative cardiac patients. If the true RQ increases, then the VO_2_ would be overestimated and the calculated CAPNO-SvO_2_ would be too low (see Eqs. 1 and 3). Thus, the CAPNO-SvO_2_ will demonstrate a low SvO_2_, perhaps too low, when there is a risk for insufficient oxygen delivery and anaerobic metabolism. This is illustrated for various SvO_2_ and RQ settings in the Additional file 1, Figure S10.

Finally, the agreement between CAPNO-SvO_2_ and PAC-SvO_2_ was preserved during changes in PEEP and hence different lung conditions. The changes induced by increasing PEEP were modest and well tolerated without inducing any major cardiorespiratory changes. The purpose was to investigate the effect on EPBF and CAPNO-SvO_2_ in a situation when lung recruitment could occur that would change the pulmonary shunt. Since the VCO_2_ in the CAPNO-SvO_2_ equation is determined by the blood flow participating in gas exchange (EPBF), its robust performance should be maintained in patients with more severe lung pathology compared to the postoperative patients in this study. Future validation studies of CAPNO-SvO_2_ are needed to establish this. The results of this study can reasonably be extended as valid for elective, fasting surgical patients admitted to ICU following major procedures, but do not demonstrate that CAPNO-SvO_2_ can replace PAC-SvO_2_ for all types of ICU patients.

### Effective pulmonary blood flow by the capnodynamic method

The effective pulmonary blood flow (EPBF) represents the non-shunted fraction of the cardiac output and is, therefore, a major determinant of gas exchange and systemic oxygen delivery. Maintaining adequate oxygen delivery and timely interventions to mitigate potential reductions in organ oxygenation are important to avoid postoperative organ dysfunction, including acute kidney injury, which remains an important contributor to poor outcomes in cardiac surgery [24, 25]. The nearly continuous monitoring of EPBF by the capnodynamic method is suited to enable prompt recognition and treatment of a low cardiac output state postoperatively.

In this study, the percentage error between EBPF and CO_TD_ was slightly higher than that for SvO_2_ but remained below the 30% limit stated by Critchley and Critchley for interchangeability with CO_TD_ [17]. The bias observed was similar to previous reports, even though the presence of non-proportionality was not reported in these earlier studies, and within the usual limits of exclusion (< 15%) in concordance analyses where CO_TD_ is used as reference [12, 26–28]. The systematic bias is not surprising since the two variables are not identical physiological entities (EPBF = CO–shunt flow). The observed overall bias of –0.41 l.min^−1^ in this study corresponds to a 10% relative difference, which plausibly represents an average shunt fraction of 10% in the study cohort. In contrast to CO_TD_, the EPBF increased after the PEEP-manoeuvre, likely reflecting a reduction of the shunt with opening of atelectatic lung areas. The divergence between EPBF and CO in the presence of atelectasis has been reported previously [7, 27]. The low percentage error found in this study suggests that EPBF is clinically acceptable for monitoring cardiac output. This is also supported by a recent publication where both EPBF and CAPNO-SvO_2_ were able to monitor low cardiac output states and the haemodynamic effects of intravascular blood volume changes in a pig model of standardised haemorrhage and subsequent volume resuscitation [6, 10].

Both CAPNO-SvO_2_ and EPBF are derived from the same measure, VCO_2_, and the differences in agreement analyses (bias, limits of agreement and percentage error) are likely related to the different clinical reference methods used. The accuracy of blood gas analysis, if the blood sample is properly handled, is superior to thermodilution measurements of CO, which are more variable [12, 29].

### Sources of error in the capnodynamic method and study limitations

An obvious limitation of the capnodynamic method is the requirement for controlled mechanical ventilation with satisfactory patient-ventilator synchrony. Data quality deteriorates and might become uninterpretable in the presence of spontaneous breathing efforts. The capnodynamic software features an integrated control function that alerts and allows the user to discard measurements of uncertain data quality during spontaneous breathing activity (based on variation in breath duration, apparent breath-by-breath respiratory compliance, and the quality of fit between measured data and the dynamic model equations). All patients in this study were without spontaneous breathing activity with residual intraoperative neuromuscular blockade present in most.

The calculated EPBF is inversely proportional to the solubility of CO_2_ in blood, which depends on the haemoglobin concentration used in the content equations [5]. An incorrectly high haemoglobin of 10 g.l^−1^ above the true value would result in an underestimation of EPBF by 4%. This would have two counteracting effects on CAPNO-SvO_2_: while EPBF would be underestimated, the oxygen-carrying capacity of blood would be overestimated (see Methods, Eq. 2), resulting in a true SvO_2_ of 70% reported as 71.1%. Since VCO_2_ applies to both the numerator and the denominator of the CAPNO-SvO_2_ equation (see Methods, Eq. 3), any error in determining VCO_2_ is unlikely to significantly affect CAPNO-SvO_2_. The presence of any postoperative tricuspid regurgitation was not formally assessed and might represent a source of error in the thermodilution measurements used as clinical standard reference.

The study dataset comprised 185 paired observations compared to the 191 estimated in the power calculation, corresponding to a power of 0.88 that remained very close to the target 0.9 (see Additional File 1, Figure S1). Study enrolment was not consecutive, and patients were sufficiently haemodynamically stable to tolerate the recruitment manoeuvre, meaning that a degree of spectrum bias cannot be excluded. The study was designed to evaluate agreement between the capnodynamic method and a clinical standard. The inherent precision and least significant changes for CAPNO-SvO_2_ and PAC-SvO_2_ in previous experimental studies [5, 6] indicated a concordance exclusion zone about 7% points. The PEEP-steps in this agreement study did not induce sufficient changes beyond this value (in only 11 out of 138 measurements did PAC-SvO_2_ change > 6%, data not shown), and a concordance analysis of changes was hence not meaningful since most changes would fall within the exclusion zone [30]. The capnodynamic method has performed well for trending ability in experimental studies [5, 6] and in a recent paediatric clinical study [31]. Trending data are lacking in this study of stable postoperative patients with moderate fluctuations in the study variables, and needs to be established in critically ill adult patients with sufficient variability in cardiorespiratory function. The correlation between CAPNO-SvO_2_ and PAC-SvO_2_ (*r* = 0.75) as well as between EPBF and CO_TD_ (*r* = 0.83) for changes with the greatest magnitude do, however, lend support to the capnodynamic method to track changes in the clinical reference variables (see Additional file 1, Figure S11).

## Conclusion

The results of this study in haemodynamically stable postoperative cardiac patients admitted to ICU support the study agreement hypothesis of < 10 percentage points difference between the capnodynamic algorithm to calculate mixed venous oxygen saturation and the reference blood gas analyses using a pulmonary artery catheter. The effective pulmonary blood flow agreed with thermodilution cardiac output, but the agreement was proportionally influenced by the presence of any pulmonary shunt flow. The trending ability of the capnodynamic method to capture significant changes and its use in other ICU patients with cardiorespiratory compromise warrant further investigation. The capnodynamic method provides key and timely, near-continuous information on systemic oxygen balance with minimal change to standard clinical equipment, and without requiring any particular user expertise.

## Supplementary Information

Supplementary Material 1. Contains the STARD checklist; analyses of intra-individual variation; power calculation; parametric analysis, frequency distribution, density distributionand Bland–Altman assumption checksfor the CAPNO-SvO_2_ vs. PAC-SvO_2_ data; frequency distribution, density distributionand Bland–Altman assumption checksfor the EPBF vs. CO_TD_ data; repeated measures ANOVA; impact of different RQ set values in the capnodynamic algorithm; and correlations of changes in mixed venous oxygen saturation and perfusion.

## Data Availability

Study data are available upon reasonable request and as allowed by HREC regulations after contact with the corresponding author.

## Abbreviations

CAPNO-SvO_2_: Mixed venous oxygen saturation by capnometry
CcO_2_: Contents of oxygen in pulmonary capillary blood
CO_TD_: Cardiac output by thermodilution
CvO_2_: Contents of oxygen in mixed venous blood
EPBF: Effective pulmonary blood flow
PAC-SvO_2_: Mixed venous oxygen saturation by pulmonary arterial catheter blood sampling and gas analysis
PEEP: Positive end-expiratory pressure
RQ: Respiratory quotient
SD: Standard deviation
VCO_2_: Carbon dioxide production
VO_2_: Oxygen consumption
95% CI: 95% Confidence Interval
