# Supplementary material for: Validation of the capnodynamic method to calculate mixed venous oxygen saturation in postoperative cardiac patients
Source: Intensive Care Med Exp. 2025 Mar 7;13:32. doi: 10.1186/s40635-025-00741-z (PMC11889286; doi:10.1186/s40635-025-00741-z)
Supplement: Supplementary file 1 — Supplementary Material 1. Contains the STARD checklist; analyses of intra-individual variation; power calculation; parametric analysis, frequency distribution, density distributionand Bland–Altman assumption checksfor the CAPNO-SvO2 vs. PAC-SvO2 data; frequency distribution, density distributionand Bland–Altman assumption checksfor the EPBF vs. COTD data; repeated measures ANOVA; impact of different RQ set values in the capnodynamic algorithm; and correlations of changes in mixed venous oxygen saturation and perfusion. [file 40635_2025_741_MOESM1_ESM.docx]

Additional File 1

**Validation of the capnodynamic method to calculate mixed venous oxygen saturation and effective pulmonary blood flow in postoperative cardiac patients**

Mats Wallin, Magnus Hallback, Hareem Iftikhar, Elise Keleher, Anders Aneman

Correspondence to: Anders Aneman, Liverpool Hospital ICU

Email: anders.aneman@health.nsw.gov.au

Contents

[STARD checklist 2](#_Toc190858452)

[Statistical software 4](#_Toc190858453)

[Analyses of intra-individual variation 4](#_Toc190858454)

[Power calculation 5](#_Toc190858455)

[Parametric Bland-Altman analysis CAPNO-SvO_2_ vs. PAC-SvO_2_ 6](#_Toc190858456)

[Frequency distribution of CAPNO-SvO_2_ vs. PAC-SvO_2_ values 7](#_Toc190858457)

[Density distribution of CAPNO-SvO_2_ vs. PAC-SvO_2_ differences 8](#_Toc190858458)

[Assumption checks for the Bland-Altman analysis of CAPNO-SvO_2_ vs. PAC-SvO_2_ 9](#_Toc190858459)

[Frequency distribution of EPBF vs. CO_TD_ values 10](#_Toc190858460)

[Density distribution of EPBF vs. CO_TD_ differences 11](#_Toc190858461)

[Assumption checks for the Bland-Altman analysis of EPBF vs. CO_TD_ 12](#_Toc190858462)

[Repeated measures analysis of variance 13](#_Toc190858463)

[Impact of different RQ settings 14](#_Toc190858464)

[Correlation of changes in mixed venous saturation and perfusion 15](#_Toc190858465)

# STARD checklist

**Table S1.** STARD checklist [7, 8].

|  | **Section & Topic** | **No** | **Item** | **Reported on page #** |  |
| --- | --- | --- | --- | --- | --- |
|  | |  |  |  |  |
|  | | **TITLE OR ABSTRACT** |  |  |  |
|  | |  | **1** | Identification as a study of diagnostic accuracy using at least one measure of accuracy  (such as sensitivity, specificity, predictive values, or AUC) | 1 |
|  | | **ABSTRACT** |  |  |  |
|  | |  | **2** | Structured summary of study design, methods, results, and conclusions  (for specific guidance, see STARD for Abstracts) | 2 |
|  | | **INTRODUCTION** |  |  |  |
|  | |  | **3** | Scientific and clinical background, including the intended use and clinical role of the index test | 3,4 |
|  | |  | **4** | Study objectives and hypotheses | 4 |
|  | | **METHODS** |  |  |  |
|  | | *Study design* | **5** | Whether data collection was planned before the index test and reference standard  were performed (prospective study) or after (retrospective study) | 5 |
|  | | *Participants* | **6** | Eligibility criteria | 5 |
|  | |  | **7** | On what basis potentially eligible participants were identified  (such as symptoms, results from previous tests, inclusion in registry) | 4 |
|  | |  | **8** | Where and when potentially eligible participants were identified (setting, location and dates) | 4 |
|  | |  | **9** | Whether participants formed a consecutive, random or convenience series | 5 |
|  | | *Test methods* | **10a** | Index test, in sufficient detail to allow replication | 8 |
|  | |  | **10b** | Reference standard, in sufficient detail to allow replication | 8 |
|  | |  | **11** | Rationale for choosing the reference standard (if alternatives exist) | 2,8 |
|  | |  | **12a** | Definition of and rationale for test positivity cut-offs or result categories  of the index test, distinguishing pre-specified from exploratory | 9, Fig S1 |
|  | |  | **12b** | Definition of and rationale for test positivity cut-offs or result categories  of the reference standard, distinguishing pre-specified from exploratory | 9, Fig S1 |
|  | |  | **13a** | Whether clinical information and reference standard results were available  to the performers/readers of the index test | 8 |
|  | |  | **13b** | Whether clinical information and index test results were available  to the assessors of the reference standard | 8 |
|  | | *Analysis* | **14** | Methods for estimating or comparing measures of diagnostic accuracy | 9 |
|  | |  | **15** | How indeterminate index test or reference standard results were handled | 9 |
|  | |  | **16** | How missing data on the index test and reference standard were handled | 9 |
|  | |  | **17** | Any analyses of variability in diagnostic accuracy, distinguishing pre-specified from exploratory | 9 |
|  | |  | **18** | Intended sample size and how it was determined | 9 |
|  | | **RESULTS** |  |  |  |
|  | | *Participants* | **19** | Flow of participants, using a diagram | 10, Fig 1 |
|  | |  | **20** | Baseline demographic and clinical characteristics of participants | 10, Table 1 |
|  | |  | **21a** | Distribution of severity of disease in those with the target condition | Suppl. 4,5 and 7.8 |
|  | |  | **21b** | Distribution of alternative diagnoses in those without the target condition | Suppl. 4,5 and 7.8 |
|  | |  | **22** | Time interval and any clinical interventions between index test and reference standard | 9 |
|  | | *Test results* | **23** | Cross tabulation of the index test results (or their distribution)  by the results of the reference standard | 10 |
|  | |  | **24** | Estimates of diagnostic accuracy and their precision (such as 95% confidence intervals) | 10 |
|  | |  | **25** | Any adverse events from performing the index test or the reference standard | NA |
|  | | **DISCUSSION** |  |  |  |
|  | |  | **26** | Study limitations, including sources of potential bias, statistical uncertainty, and generalisability | 15 |
|  | |  | **27** | Implications for practice, including the intended use and clinical role of the index test | 15, 16 |
|  | | **OTHER INFORMATION** |  |  |  |
|  | |  | **28** | Registration number and name of registry | NA |
|  | |  | **29** | Where the full study protocol can be accessed | 4 |
|  | |  | **30** | Sources of funding and other support; role of funders | NA |
|  | |  |  |  |  |

# Statistical software

The statistical analyses were performed using the R programming language for statistical computing and data visualisation running in RStudio with the ‘dplyr’, ‘ggplot2’, ‘SimplyAgree’, ‘lme4’, ‘stats’, ‘emmeans’ and ‘ega' packages.

# Analyses of intra-individual variation

**Table S2.** Variance (mean ± standard deviation) for all measurements at all steps in the study protocol (top, shaded row) and coefficients of variation (CV) for each step in the study protocol during the alveolar recruitment manoeuvre (n=43).

| VARIANCE | CAPNO-SvO_2_ | ABG-SvO_2_ | EPBF | CO_TD_ |
| --- | --- | --- | --- | --- |
|  | 38.1 ± 6.2 | 30.0 ± 5.5 | 0.68 ± 0.8 | 0.76 ±0.9 |
| CV | CAPNO-SvO_2_ | ABG-SvO_2_ | EPBF | CO_TD_ |
| PRE_RM_ | 0.11 | 0.08 | 0.24 | 0.21 |
| PRE_RM_ +5 cm H_2_O | 0.10 | 0.10 | 0.21 | 0.21 |
| PRE_RM_ +10 cm H_2_O | 0.10 | 0.10 | 0.22 | 0.22 |
| POST_RM_ = PRE_RM_ | 0.10 | 0.09 | 0.24 | 0.26 |

# Power calculation

**Figure S1.** Bland-Altman analyses in previous experimental studies of CAPNO-SvO2 against PAC-SvO2 [5,6] have demonstrated minimal bias (-1 to 3 percentage points) with a standard deviation for the limits of agreement between 5-6 percentage points. In the Bland-Altman analysis power calculation for this study, the bias was set to 2 percentage points with the standard deviation for the limits of agreement set to 5 percentage points. We aimed for a confidence level of 95% to detect an agreement level of 0.8 for the hypothesised difference of ±10% and estimated that 191 data pairs were needed for a power >0.9 (blue solid line). The actual power achieved from 185 observations is shown by the dotted red line.

# Parametric Bland-Altman analysis CAPNO-SvO_2_ vs. PAC-SvO_2_

**Figure S2.** A sensitivity analysis removing one outlier and using a parametric repeated measurements Bland-Altman analysis was performed. The bias was -0.05 % [95%CI -0.93 – 0.82] (middle purple band) with the upper limit of agreement at 8.3 % [95%CI 7.2 – 9.5] (upper blue band) and the lower limit of agreement at -8.4 % [95%CI -9.6 – -7.4] (bottom yellow band).


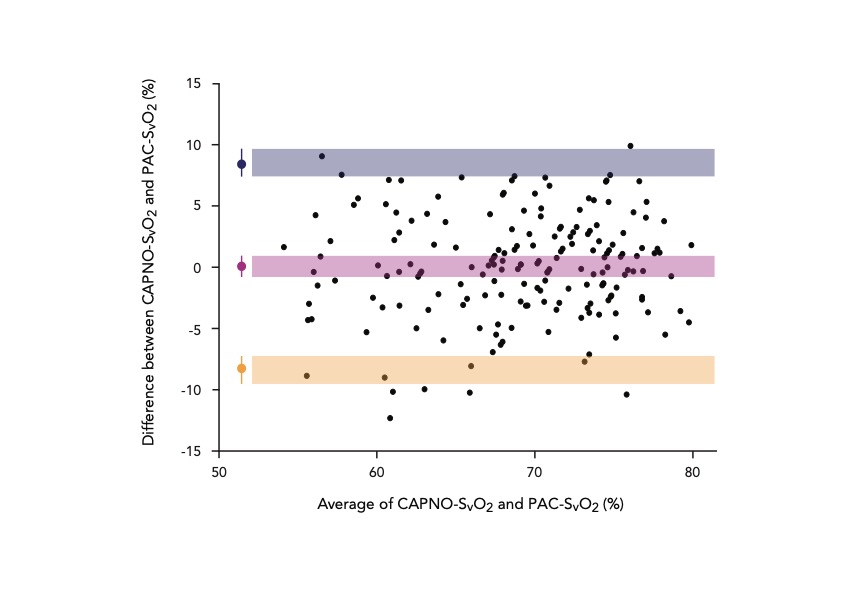


# Frequency distribution of CAPNO-SvO_2_ vs. PAC-SvO_2_ values

**Figure S3.** The frequency distribution of CAPNO-SvO_2_ (blue colour) and PAC-SvO_2_ (orange colour) values with the normal distributions overlaid and the standard deviations shown.

# Density distribution of CAPNO-SvO_2_ vs. PAC-SvO_2_ differences

**Figure S4.** The density distribution of differences between CAPNO-SvO_2_ and PAC-SvO_2_ with the normal distribution shown by the red line.

# Assumption checks for the Bland-Altman analysis of CAPNO-SvO_2_ vs. PAC-SvO_2_

**Figure S5.** Normality of residuals (top), homogeneity of variance (middle) and proportional bias (bottom) assessments. Statistical tests and p-values are listed for each graph.

# Frequency distribution of EPBF vs. CO_TD_ values

**Figure S6.** The frequency distribution of EPBF (blue colour) and CO_TD_ (orange colour) values with the normal distributions overlaid and the standard deviations shown.

# Density distribution of EPBF vs. CO_TD_ differences

**Figure S7.** The density distribution of differences between EPBF and CO_TD_ with the normal distribution shown by the red line.

# Assumption checks for the Bland-Altman analysis of EPBF vs. CO_TD_

**Figure S8.** Normality of residuals (top), homogeneity of variance (middle) and proportional bias (bottom) assessments. Statistical tests and p-values are listed for each graph.

# Repeated measures analysis of variance

**Figure S9.** Repeated measures ANOVA for mixed venous oxygen saturation derived from the capnodynamic algorithm (CAPNO-SvO_2_) and by co-oximetry of blood aspirated from the pulmonary artery catheter (PAC-SvO_2_), effective pulmonary blood flow (EPBF) and thermodilution cardiac output (CO_TD_). Two-sided p-values are given below variable names.

# Impact of different RQ settings

**Figure S10.** The impact of different set values for the respiratory quotient (RQ, x-axis) in the capnodynamic algorithm to calculate mixed venous oxygen saturation (CAPNO-SvO_2_, y-axis). The numerical values for RQ=0.7 and RQ=1.0 are shown next to the lines for a CAPNO-SvO_2_ of 0.7 (•), 0.65 (◼), 0.60 (▲) and 0.55 (▼) using the default RQ of 0.85 used in this study (dashed vertical line). An increased RQ potentially associated with anaerobic glycolysis would generate a higher CAPNO-SvO_2_, meaning that the study RQ of 0.85 could indicate a lower SvO_2_, but not a spuriously higher value that might delay corrective therapeutic measures.

# Correlation of changes in mixed venous saturation and perfusion

**Figure S11.** Correlation between mixed venous oxygen saturation derived from the capnodynamic algorithm (CAPNO-SvO_2_) and by co-oximetry of blood aspirated from the pulmonary artery catheter (PAC-SvO_2_), effective pulmonary blood flow (EPBF) and thermodilution cardiac output (CO_TD_). The magnitude of changes (Δ) induced by the largest variation in PEEP (baseline pre-recruitment (PRE_RM_) vs. after an additional 10 cm H_2_O PEEP (PRE_RM_ + 10) and after the return to baseline post-recruitment from PRE_RM_ +10) were analysed. The hashed rectangle for SvO_2_ measurements indicates the exclusion zone at 6% based on the least significant change for PAC-SvO_2_ [5]. The correlations are reported by Pearson correlation coefficient (r) including the 95% confidence interval [95%CI].
